# Safe and effective subcutaneous adipolysis in minipigs by a collagenase derivative

**DOI:** 10.1371/journal.pone.0227202

**Published:** 2019-12-31

**Authors:** Fuding Chen, Gang Du, Meishu Shih, Hongjiang Yuan, Peng Bao, Sheng Shi, Yong Cang, Zhen Zhang

**Affiliations:** 1 Research and Development Division, Rejuven Dermaceutical Co., Ltd., Hangzhou, China; 2 PharmaLegacy Laboratories (Shanghai) Co., Ltd., Shanghai, China; Government College University Faisalabad, PAKISTAN

## Abstract

Adipocytes attached to the extracellular matrix (ECM) mainly consist of collagen in adipose tissues, while the degradation of ECM by collagenase induces the apoptosis of adipocytes, leading to a decrease in local subcutaneous adipose. To achieve this goal, we are developing a mutant collagenase H (ColH) to remove local subcutaneous fat such as submental fat (SMF). Three vectors were constructed for expressing rColH(FM, mutant for fat melting, with 6xHis tag), rColH(WT, wild-type, with 6xHis tag), and rColH(E451D, E451D mutant, without 6xHis tag) in *Escherichia coli*. rColH(FM) & rColH(WT) were purified by Ni Sepharose on a laboratory scale, while rColH(E451D) was purified by five chromatography purification steps on a large scale. Then, the stability of rColH(FM) and rColH(WT) was tested by SDS-PAGE to investigate the influence of the E451D mutation on stability. Afterwards, the enzyme kinetics of ColH (mutant or wild-type, with or without His tag) were investigated and compared. Finally, the adipolysis of rColH(E451D) at various doses was tested *in vitro* and *in vivo*. The ultrasound results in minipigs suggested that effective adipolysis was induced by rColH(E451D) compared with the negative control, and the histological results suggest dose-dependent fibrosis, necrosis, inflammation and cholesterol cleft formation. These findings indicate the possibility of rColH(E451D) becoming a new injectable drug to safely remove subcutaneous adipose.

## Introduction

Collagenase from *Clostridium histolyticum* was first isolated and reported in 1953 [1]. Thirty years later, six collagenase subtypes (α, β, γ, δ, ε, and ζ) were separated and characterized in detail [2–4]. Compared with Type I collagenases, collagenase H (ζ subtype), a Type II collagenase, has increased activity on synthetic peptide substrates. These two types of collagenases are both metalloproteinases that can digest collagen, the main component of the ECM in animal tissues [5]. Due to the specific collagenolytic properties of collagenase from *C*. *histolyticum*, clostridial collagenase was used to treat tissues from patients with Peyronie's disease [6] and was injected into Dupuytren's cords from patients undergoing fasciotomy [7]. Subsequently, *C*. *histolyticum* collagenase (Xiaflex®) was approved by the FDA to treat Dupuytren's disease in 2010 and Peyronie's disease in 2013. In addition, collagenases are widely applied in therapeutic fields such as wound healing, debridement [8, 9] and burn, cellulite and uterine fibroid treatment [10]. In particular, collagenase H (ColH) is crucial for the isolation of rat pancreatic islets [11]. Also, collagenase has been used in the isolation of adipocytes from adipose tissues [12–14]. Recently, collagenase was applied in aesthetic medicine by physicians to dissociate human adipose tissues and increase the viability of human adipose cells for autologous fat transplantation [15, 16].

Localized adipose accumulation, such as SMF and cellulite, makes some people feel dissatisfied with their bodies. Removal of unwanted “double chin” may be of particular interest for these people. The traditional treatment for SMF is surgery, such as liposuction. However, surgery is invasive and may have apparent risks to patients, including extended recovery time, infection, anesthesia, and bruising and scarring at surgical sites [17, 18]. Other than liposuction, minimally invasive treatments such as laser-assisted lipolysis and radio frequency–assisted (RF) lipolysis were used to reduce local adipose tissues with fewer adverse effects [19, 20]. Given the risks of surgery, noninvasive therapy is desirable, and such treatments, including deoxycholic acid (Kybella®) [18] and cryolipolysis (CoolMini) [21], have been developed to reduce local adipose tissues such as SMF. Deoxycholic acid is a surfactant with a structure similar to cholesterol, so it is able to disrupt cell membranes and induce the lysis of adipocytes after injection into adipose tissues [18].

In this study, our objective was to investigate an alternate noninvasive method for the adipolysis of local adipose tissues, which is the first time for collagenase to be applied to this area. According to previous studies, collagenase could digest ECM and liberate adipocytes *in vitro*; however, the wild-type collagenase may cause severe side effects *in vivo*, such as hemorrhage. For our study, an E451D mutant ColH (rColH(E451D)) expressed in *E*. *coli* was purified and investigated. The kinetic analysis results we obtained were similar to those of a previous study [22]. The data indicate that the E451D mutation of ColH does not impair its substrate (i.e., collagen) binding function but leads to the slower digestion of collagen. Furthermore, adipolysis tests were conducted *in vitro* and *in vivo* to investigate the efficacy and safety of rColH(E451D).

## Materials and methods

### Bacterial strains, plasmids, and construction

**Fig 1:**
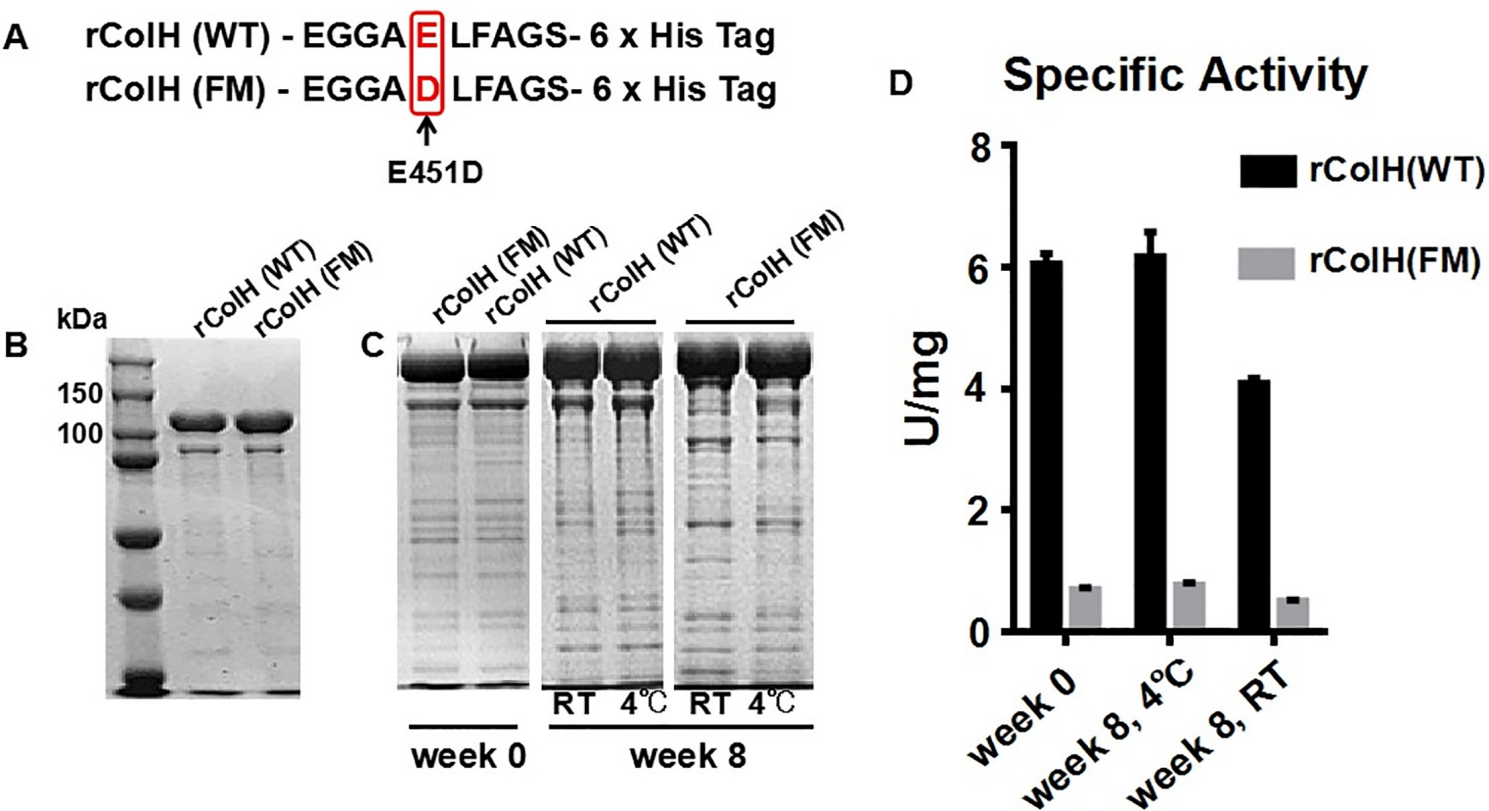
Stability analysis of rColH(WT) and rColH(FM). (A) rColH(FM) is a mutant rColH(WT) with an E451D mutation, and both contain His tag at the C-terminus. (B) SDS-PAGE analysis of rColH(WT) and rColH(FM) after NTA-Ni chromatography purification. (C) SDS-PAGE analysis of rColH(FM) and rColH(WT) before and after storage at RT or 4°C for 8 weeks. (D) Specific activities of rColH(FM) and rColH(WT) before and after storage at RT or 4°C for 8 weeks. Data are presented as the mean ± standard deviation (SD), n = 3.

*coli* BL21(DE3) was obtained from Merck (USA) as host cells for expressing ColH, and pET-30a(+) plasmid DNA was obtained from Merck (USA) as an expression vector. pET-30a(+) plasmids containing synthetic *colH(WT/FM)* genes were obtained from General Biosystems Inc. (Chuzhou, Anhui, China) and transformed into *E*. *coli* BL21(DE3) by heat shock to express rColH(WT) or rColH(FM), both of which contain 6×His tag at the C-terminus. rColH(FM) is a mutant of rColH(WT) with E451D (Fig 1A). The *colH(E451D)*gene was amplified by PCR and constructed into pET-30a(+), which was then transformed into *E*. *coli* BL21(DE3) by heat shock to express rColH(E451D), which does not contain 6×His tag. As a consequence, rColH(E451D) can be regarded as a mutant of United States Pharmacopoeia Collagenase II (USP ColH) on E451D. *Nde* I & *Xho* I restriction enzymes and T4 DNA ligase were purchased from Thermo (USA); protein molecular weight markers were purchased from Bio-Rad (USA). The following primers were used for the amplification of the *colH(E451D)* gene: ColH-F, 5’-GGGAATTCCATATGGTTCAGAATGAGAGTAAACG-3’ and ColH-R, 5’-CCGCTCGAGTTAGCGACCCACGCTACCTTCAATG-3’ (the recognition sites of the restriction enzymes are underlined).

### Media and culture conditions

The medium used to prepare rColH(WT)/rColH(FM) and for the seed culture of rColH(E451D) was LB medium. Fed-batch cultivation was used to prepare rColH(E451D) and was carried out in a 65-L bioreactor (NBS) with 30 L of fermentation medium consisting of 405 g of tryptone, 210 g of yeast extract, 45 g of ammonium sulfate, 120 g of sodium chloride, 12 g of anhydrous magnesium sulfate, 12 mL of defoamer, 315 g of disodium hydrogen phosphate dodecahydrate, 90 g of potassium dihydrogen phosphate and 180 g of dipotassium hydrogen phosphate. A concentrated glucose solution (300 g of glucose in 1 L) was added as a carbon source before inoculation. A 7.5 L solution of feeding medium was composed of 1500 g of glycerol, a total of 525 g of yeast extract, 1050 g of tryptone and 54 g of anhydrous magnesium sulfate. Diluted phosphoric acid and ammonia solution were added to adjust the pH. Tryptone and yeast extract were from OXOID, and others were from Sinopharm Chemical Reagent Co., Ltd. (Shanghai, China).Protein expression was induced by 0.5 mM isopropyl-β-d-thiogalactopyranoside (IPTG) for 4 h. The dissolved oxygen level was maintained at 30% by varying the agitation rate and feeding rate.

### Purification of wild-type and mutant ColH proteins

After cultivation and expression, the cells were collected by centrifugation at 5,000 rpm and 4°C for 20 minutes. The cells were resuspended in Buffer A (100 mM Tris-HCl, pH 7.5) and lysed by ultrasound (for rColH(WT) & rColH(FM)) or homogenization (for rColH(E451D)). The supernatant of the cell lysate was loaded into a column of Ni Sepharose High Performance (GE Healthcare Life Sciences) for the purification of rColH(WT) & rColH(FM). After washing with washing buffer (Buffer A + 50 mM imidazole, pH 7.5), rColH(WT) or rColH(FM) was eluted with elution buffer (Buffer A + 150 mM imidazole, pH 7.5).

The purification of rColH(E451D) was conducted on an AKTA pilot system (GE) through five chromatography steps. 1) The supernatant was loaded into a Capto Phenyl HS column that had been equilibrated with Buffer B (Buffer A + 1.2 M (NH_4_)_2_SO_4_, pH 7.5). The protein was eluted with Buffer A after being washed with Buffer C (Buffer A + 0.5 M (NH_4_)_2_SO_4_, pH 7.5). 2) The eluent of Capto Phenyl HS was diluted twice and applied to a Capto Q column equilibrated with Buffer D (50 mM Tris-HCl, pH 7.5). The enzyme was washed with Buffer E (Buffer D + 50 mM NaCl, pH 7.5) and eluted with Buffer F (Buffer D + 100 mM NaCl, pH 7.5). 3) The conductivity for the eluent of Capto Q was adjusted by Buffer G (Buffer A + 2 M (NH_4_)_2_SO_4_, pH 7.5); then, it was loaded into a Capto Octyl column equilibrated with Buffer B. After washing with Buffer H (Buffer A + 1 M (NH_4_)_2_SO_4_, pH 7.5), the enzyme was eluted with Buffer I (Buffer A + 0.5 M (NH_4_)_2_SO_4_, pH 7.5). 4) The conductivity for the eluent of Capto Octyl was adjusted by Buffer G (Buffer A + 2 M (NH_4_)_2_SO_4_, pH 7.5); then, it was loaded into a Phenyl HP column equilibrated with Buffer B. After washing with Buffer J (Buffer A + 0.5 M (NH_4_)_2_SO_4_, pH 7.5), the enzyme was eluted with Buffer K (Buffer A + 0.1 M (NH_4_)_2_SO_4_, pH 7.5). 5) The eluent from the Phenyl HP was diluted five times and then loaded into a Source 15Q column equilibrated with Buffer D. The enzyme was washed with Buffer L (Buffer D + 50 mM NaCl, pH 7.5) and eluted with Buffer M (Buffer D + 100 mM NaCl, pH 7.5). Capto Phenyl HS, Capto Q, Capto Octyl, Phenyl HP, and Source 15Q were purchased from GE. The stability at 4°C and room temperature (RT, 25±2°C) for 8 weeks of four kinds of proteins was investigated by SDS-PAGE analysis, and their activities were assayed.

### Purity assay and identification

The purity of wild-type and mutant ColH enzymes was first examined by SDS-PAGE. Proteins were separated on a 7.5% polyacrylamide gel and stained with Coomassie brilliant Blue R. Then, the purity was tested by size exclusion chromatography (SEC) and SDS-capillary electrophoresis (CE-SDS). SEC was performed according to USP <89.2> Collagenase II [23], and CE-SDS was performed according to Chinese Pharmacopeia 2015. Q-TOF-MS (XevoG2-XS Q-TOF, Waters) was employed to identify the molecular weight (MW) of rColH(E451D) (conducted by Shanghai Applied Protein Technology Co., Ltd.). The test time was 20 minutes, and the positive ion & parent ion scanning range was 500–4000 m/z.

### Enzyme assay

An *in vitro* enzyme activity assay was conducted according to USP <89.2> Collagenase II, using a synthetic peptide (4-phenylazobenzyloxycarbonyl (PZ)-Pro-Leu-Gly-Pro-D-Arg, trifluoroacetate salt, Sigma) as the substrate. The test substances were diluted to 0.2–0.3 mg/mL by 0.1 M Tris, pH 7.1. Transfer 1.0 mL of Substrate solution and 0.2 mL of 0.2M Calcium chloride solution into a test tube, and equilibrate the test tube to 25°C. Start the reaction by adding 0.05 mL of test article or blank of Tris buffer. Mix and incubate for exactly 15 min at 25°C. One enzyme unit will release the equivalent of 1 μmol of PZ-Pro-Leu from PZ-Pro-Leu-Gly-Pro-D-Arg per minute under the assay conditions. The activity calculation formula refers to USP <89.2> Collagenase II. For enzyme kinetics studies, the assay was carried out with various concentrations of the substrate (0.1 to 0.5 mg/mL). The *K*_*m*_ and *k*_*cat*_ values were calculated by Lineweaver-Burk plot analysis; protein concentrations were determined by ultraviolet spectrophotometry (i.e. protein concentration = D * A280 / 1.4, D = dilution factor, 1.4 = extinction coefficient for collagenase). All assays were performed in triplicate.

### Animals and housing/husbandry conditions

Animals (Table 1) were provided by Wujiang Tianyu Biotechnology Co., Ltd. meeting PharmaLegacy’s procurement requirement of specific pathogen free. Pigs were housed in in-door animal room meeting AAALAC requirement. A health inspection was performed on each animal to include evaluation of the surface, extremities and orifices. Each animal was also examined for any abnormal signs in posture or movement.

**Table 1:** The information of animals used in this study.

Animal species and strain	Bama miniature pig
History of treatment	Naive
Gender	1 Female for *in vitro* study 2 Males and 1 Female for *in vivo* study
Age	6 months
Weight	Around 30 kg
Breeder/supplier	Wujiang Tianyu Biotechnology Co., Ltd.
Acclimation period	7–14 days

The procedures were approved by PharmaLegacy Laboratories IACUC.

Animals were group housed in pig pen. The room temperature was maintained at 16–26°C with a relative humidity of 40–70%. Illumination was fluorescent light for 12 –hour light (8:00–20:00) and 12 –hour dark.

The manufacturer (Beijing Keaoxieli Feed Co., Ltd.) supplied a certificate of analysis for each batch of diet received by PharmaLegacy Laboratories, The certificates of analysis was and will be retained in the PharmaLegacy Laboratories archives.

Water from PharmaLegacy Laboratories in house production was available to pig ad libitum throughout the study period. Water from the municipal water supply was purified by filter system and met WHO human drinking water standard. Water analysis were performed twice per year and included analyses of heavy metals, nitrates, dissolved minerals, total plate count and coliforms. Certificates of analysis was and will be retained in the PharmaLegacy Laboratories archives.

It was not anticipated that the level of known contaminants in the feed and water will interfere with the purpose or conduct of the studies.

### Efficacy on adipose tissues *in vitro*

The efficacy of rColH(FM) and rColH(E451D) *in vitro* was tested on isolated adipose tissues from *ob/ob* mice or minipigs. For the study on *ob/ob* mice’s adipose tissue, inguinal adipose tissues were dissected from *ob/ob* mice and cut into pieces by scissors. Lipolysis of adipose tissues from *ob/ob* mice was conducted on a 24-well plate. rColH(FM) at different doses was incubated with adipose tissues in a shaker at 37°C for 24 h; PBS was used as a negative control. As for minipig’s adipose tissue study, adipose tissues were dissected from the back of a drug-naive minipig after euthanasia, and were injected with 400 μL of 0.75mg/mL rColH(E451D) followed by incubation in a shaker at 37°C for 5 h. Then, the site of the injection was cut apart, and the melting oily liquid was removed. The results were visually observed and pictures were taken without any measurement.

### Efficacy on adipose tissues *in vivo*

Pharmacodynamics study of rColH(E451D) was performed on three drug-naive minipigs *in vivo*, which were purchased from Wujiang Tianyu Biotechnology Co., Ltd. (Suzhou, Jiangsu, China), and the study was conducted at PharmaLegacy Laboratories (Shanghai, China). The backs of the minipigs were divided into six areas (A-F) for subcutaneously administrating 0.075 mg of protein (A), 0.15 mg of protein (B), 0.30 mg of protein (C), placebo (D) or saline (E & F). The dosage volume is 400μL/injection. A single dose was administered at 6 injection points per 4 cm × 6 cm area, and the thickness of the adipose tissues in each area was measured weekly by ultrasonography [21]. 4 weeks after the only injection on Day 1, all the three minipigs were euthanized and the dosed sections were dissected from the back of minipigs for histology examination.

### Histological assay

Each adipose block was kept in 10% neutral buffering formalin (formaldehyde, Na_2_HPO_4_ & NaH_2_PO_4_ were purchased from Sinopharm Chemical Reagent) for at least 48 h, embedded in paraffin and sectioned into 10 μm slices. Histological analysis was based on routine hematoxylin & eosin (H & E) or Masson trichrome staining. All parameters that pathologists used to evaluate and grade were indicated by scores of 0 (normal), 1 (slight or mild), 2 (moderate), 3 (moderate to severe or marked) and 4 (marked).

### Statistical analysis

The results of the column chart are presented as the mean ± SD. All statistical analyses were performed by using a t-test on Graphpad Prism 6.0.

## Results

### Purification and stability analysis

rColH(WT) & rColH(FM) are fused with 6×His tag at the C-terminus, which facilitates the purification of proteins at the laboratory scale. Ni Sepharose affinity chromatography was employed to purify proteins in this study. After this purification, the purity of rColH(FM) & rColH(WT) was 73%-74% according to gray intensity analysis of SDS-PAGE pictures by Gel-Pro analyzer software (Fig 1B). After storage at 4°C or RT for 8 weeks, no autolysis was observed with SDS-PAGE for rColH(WT) or rColH(FM) (Fig 1C), and their enzyme activities were stable at 4°C but decreased at RT (Fig 1D). The results indicated that the E451D mutation did not influence the stability of the enzyme but resulted in lower specific activity because E451 is the key position in the catalytic domain [22]. The decrease in enzyme activity at RT after 8 weeks is reasonable due to the instability of most enzymes at RT. Since the 6×His tag in rColH(FM) is immunogenic and the purity achieved by one step of Ni-Sepharose affinity chromatography did not meet the quality requirements of clinical application, the purity of mutant collagenase needed to be improved. Subsequently, plasmids expressing rColH(E451D) without His tag were reconstructed and transformed into *E*. *coli* BL21(DE3). rColH(E451D) was developed for medical application and prepared with multiple chromatography steps.

### Preparation and identification of rColH(E451D)

**Fig 2:**
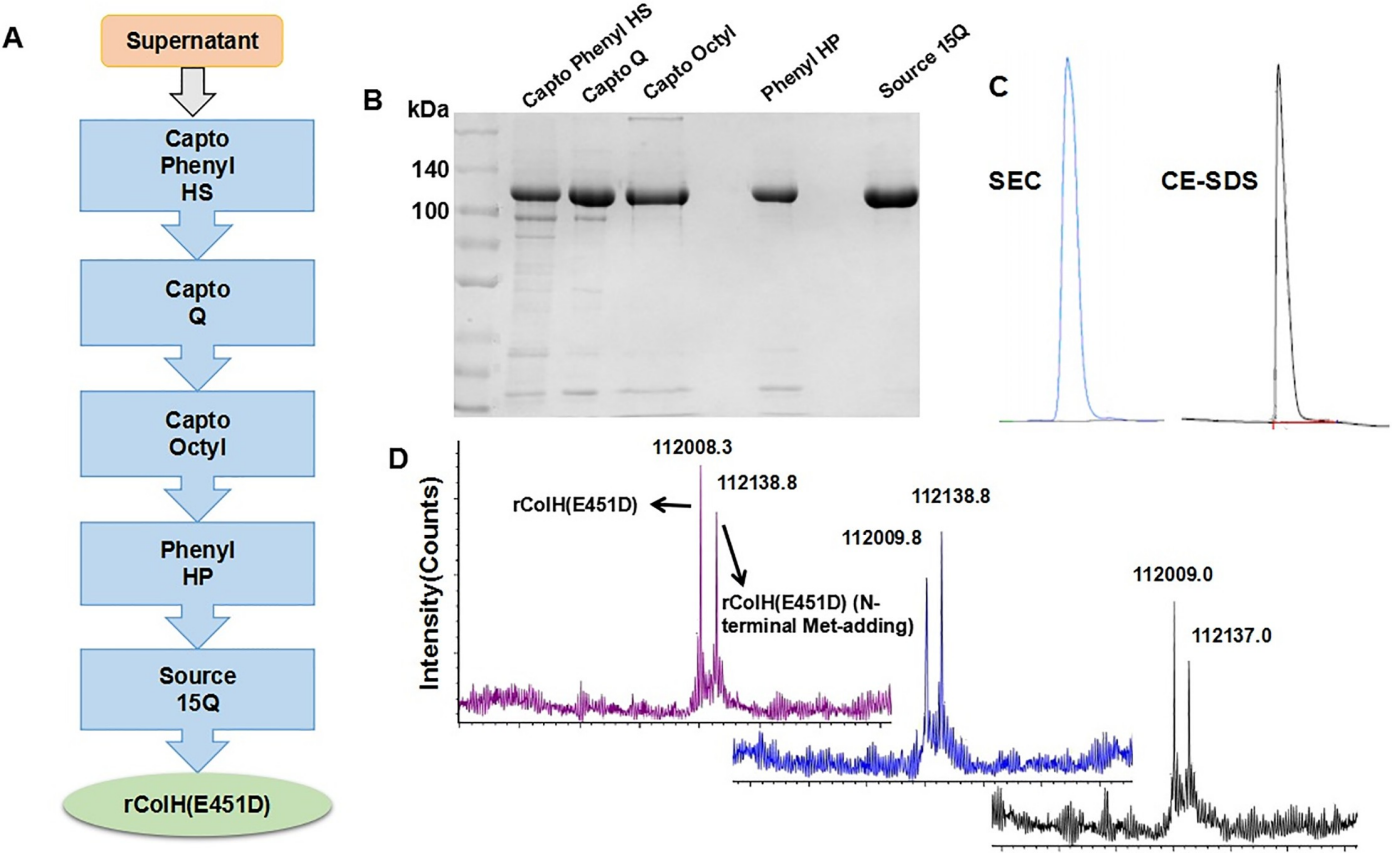
Purification and identification of rColH (E451D). (A) The process flow chart of purification with five chromatography steps. (B) SDS-PAGE of rColH(E451D) protein after five purification steps. (C) SEC and CE-SDS results of rColH(E451D) protein. (D) Q-TOF-MS of rColH(E451D) protein from three continuous batches. The theoretical molecular weight of rColH(E451D) is 112,009 Da.

To meet the requirements of druggability, His tag should not be contained in proteins. Therefore, a new strain expressing native collagenase, rColH(E451D), was prepared. rColH(E451D) is a mutant of USP ColH which is natively extracted from *C*. *histolyticum*. The strain was cultivated in a 65-L fermenter and induced by 0.5 mM IPTG. Then, the cells were harvested by centrifugation and homogenized after resuspension. The supernatant underwent five chromatography purification steps (Fig 2A) before high-purity rColH(E451D) was obtained. The eluent of each step was analyzed by SDS-PAGE (Fig 2B), and the results indicated that the purity of rColH(E451D) was continuously improved to 99% according to gray intensity analysis (Image lab, Bio-Rad). Furthermore, the purity was assessed by SEC and CE-SDS with results ≥ 98% (Fig 2C). All of the purity results suggested that rColH(E451D) met the requirements of medical application. Then, rColH(E451D) was identified by Q-TOF-MS. There were 2 main peaks (Fig 2D) with molecular weights of 112008.3 Da (rColH(E451D)) and 112138.8 Da (Met added at the N-terminus). Methionine (Met) is the first amino acid added during protein translation, and the cleavage of Met is related to the adjacent sequence [24]. Since Met is not located in the catalytic domain, the residual Met would not affect the activity of rColH(E451D).

### Kinetic analysis

**Fig 3:**
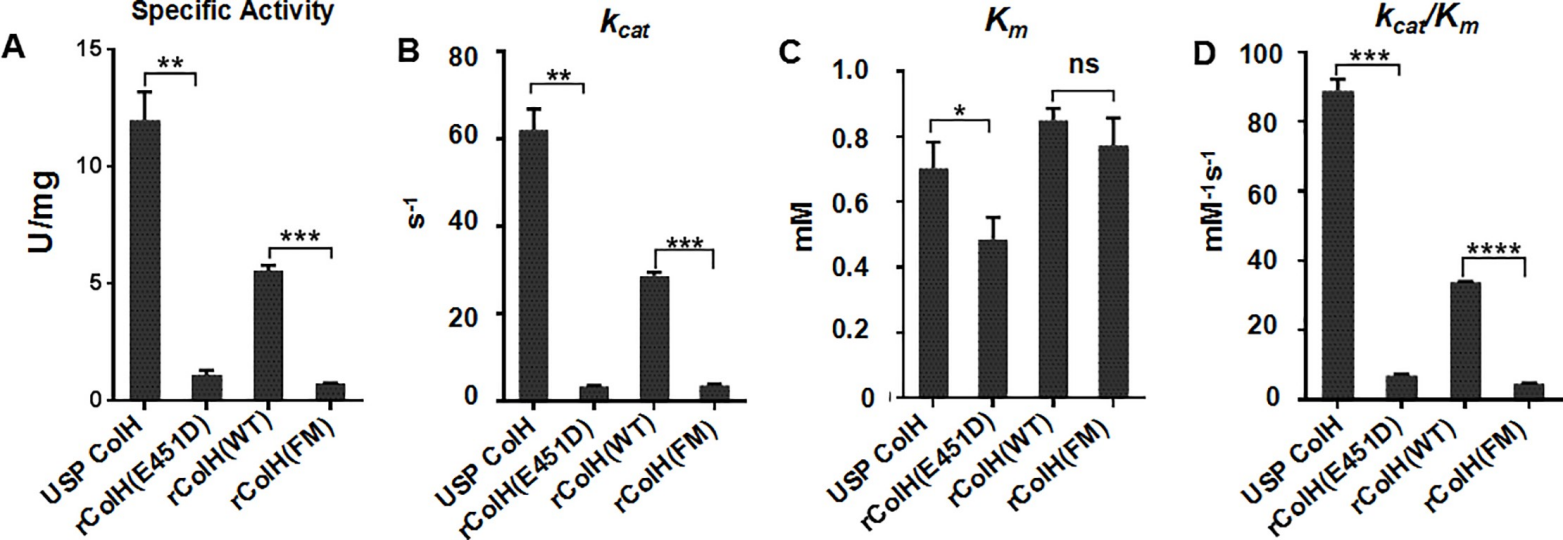
Kinetic parameters tested for USP ColH, rColH(E451D), rColH(WT) and rColH(FM). Specific activity (A), *k*_*cat*_ (B), *K*_*m*_ (C) and *k*_*cat*_/*K*_*m*_ (D) are shown in the figures. *K*_*m*_ and *k*_*cat*_ values were obtained by Lineweaver-Burk plot analysis. Values are the mean ± SD of triplicate measurements determined using Graphpad Prism 6.0. * *P<*0.05; ** *P<*0.01; *** *P<*0.001; **** *P<*0.0001. ns: not significant. The detailed data are listed in S1–S3 Tables.

To characterize the changes in enzymatic activity after the E451D mutation, we performed kinetic studies for four kinds of enzymes, including USP ColH (99%, wild-type), rColH(E451D) (99%, E451D mutant), rColH(WT) (74%, wild-type containing His tag) and rColH(FM) (73%, E451D mutant containing His tag). The specific activities of rColH(E451D) and rColH(FM) (1.1±0.19 U/mg and 0.74±0.02 U/mg, respectively) were decreased by 10.9- and 7.5-fold compared with those of USP ColH and rColH(WT), respectively. The *K*_*m*_ and *k*_*cat*_ values were calculated by the Lineweaver-Burk method. The mutation caused small differences in *K*_*m*_ and significant differences in *k*_*cat*_ (Fig 3B & 3C). The mutants’ specificity constants (*k*_*cat*_*/K*_*m*_) were reduced to approximately 10% of those of the wild-type enzymes (Fig 3D). The detailed data are listed in S1–S3 Tables. In summary, the E451D mutation does not affect the binding of the enzyme and substrate but significantly decreases the catalytic rate. Thus, enzymolysis *in vivo* will be milder, and side effects such as hemorrhage may be reduced. Refer to Supplementary files of STRENDA DB for detailed kinetics data of these four enzymes.

### *In vitro* and *in vivo* Pharmacodynamics (PD)

**Fig 4:**
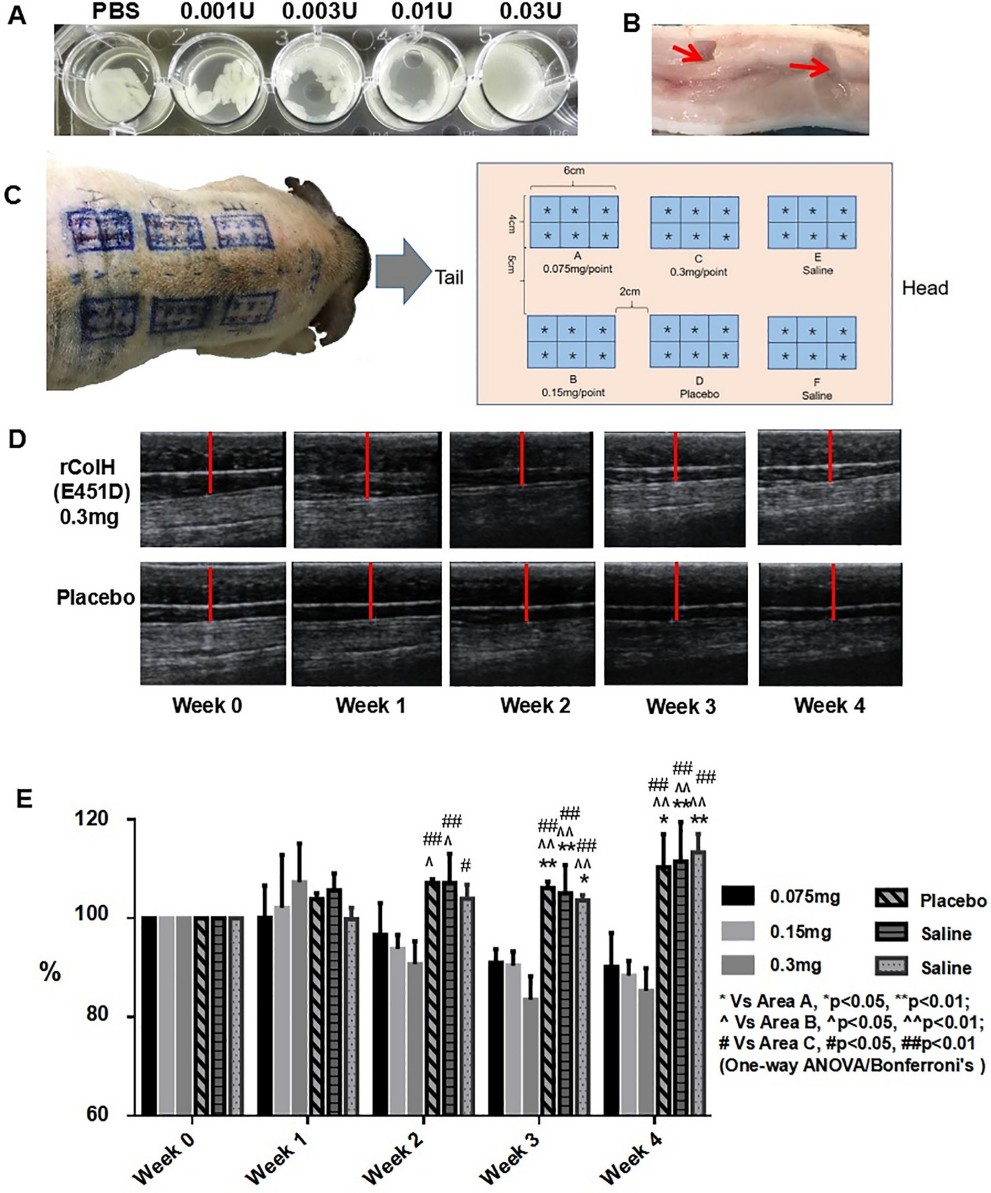
The adipolysis study *in vitro* and *in vivo*. (A) The efficacy of rColH(FM) on isolated adipose tissues from ob/ob mice was tested *in vitro*. (B) rColH(E451D) could obviously cause the adipolysis of adipose tissues from a minipig *in vitro* at 37°C in 5 h. Lipolysis was observed at injection sites after the oily liquid was removed manually (see red arrows). (C) Pharmacodynamics study schematic showing the subcutaneous injection of rColH(E451D) into the back adipose tissues of minipigs. (D) The thickness of adipose tissues was measured weekly by ultrasonography, and the results of areas C & D are shown. (E) Statistical analysis of the ultrasound results for all groups are listed. Data are presented as the mean ± SD, n = 3.

rColH(E451D) was tested both *in vitro* and *in vivo*; the results showed dose-dependent and effective lipolysis. In this study, rColH(FM) was first tested in isolated adipose tissues from ob/ob mice *in vitro* and induces dose-dependent lipolysis (Fig 4A). Then, the lipolysis of rColH(E451D) was shown *in vitro* in isolated adipose tissues from a minipig (Fig 4B). In pivotal pharmacodynamics study, three minipigs with body weights of 33±2 kg were used. As described in the methods section, three treatment groups, one placebo group and two saline groups, were established with a single dose administered on the backs of the minipigs (Fig 4C). The thickness of adipose tissues was measured weekly by ultrasonography. A portion of the typical ultrasound results is shown in Fig 4D. According to these results (Fig 4D), the thickness of adipose tissues in the group that received 0.3 mg/injection of rColH(E451D) decreased in the following weeks, while the thickness in the placebo group increased slowly due to the weight gain of the minipigs (S2 Fig). After the 4-week study, ultrasonography data in all dosing areas of three minipigs were analyzed and are shown in Fig 4E. In week 1, the thickness of the adipose tissues from the groups administered rColH(E451D) increased due to slight hematoma (Fig 4E). The results of the three rColH(E451D) groups (A, B and C) exhibited decreases in adipose tissue thickness by 9.8%, 11.6% and 14.7% following injections of 0.075, 0.15, and 0.3 mg, respectively, after 4 weeks, while those of the placebo and saline groups (D, E and F) exhibited increases of more than 10% in adipose tissue thickness due to the weight gain of the minipigs (S4 Table).

### Histological assay

**Fig 5:**
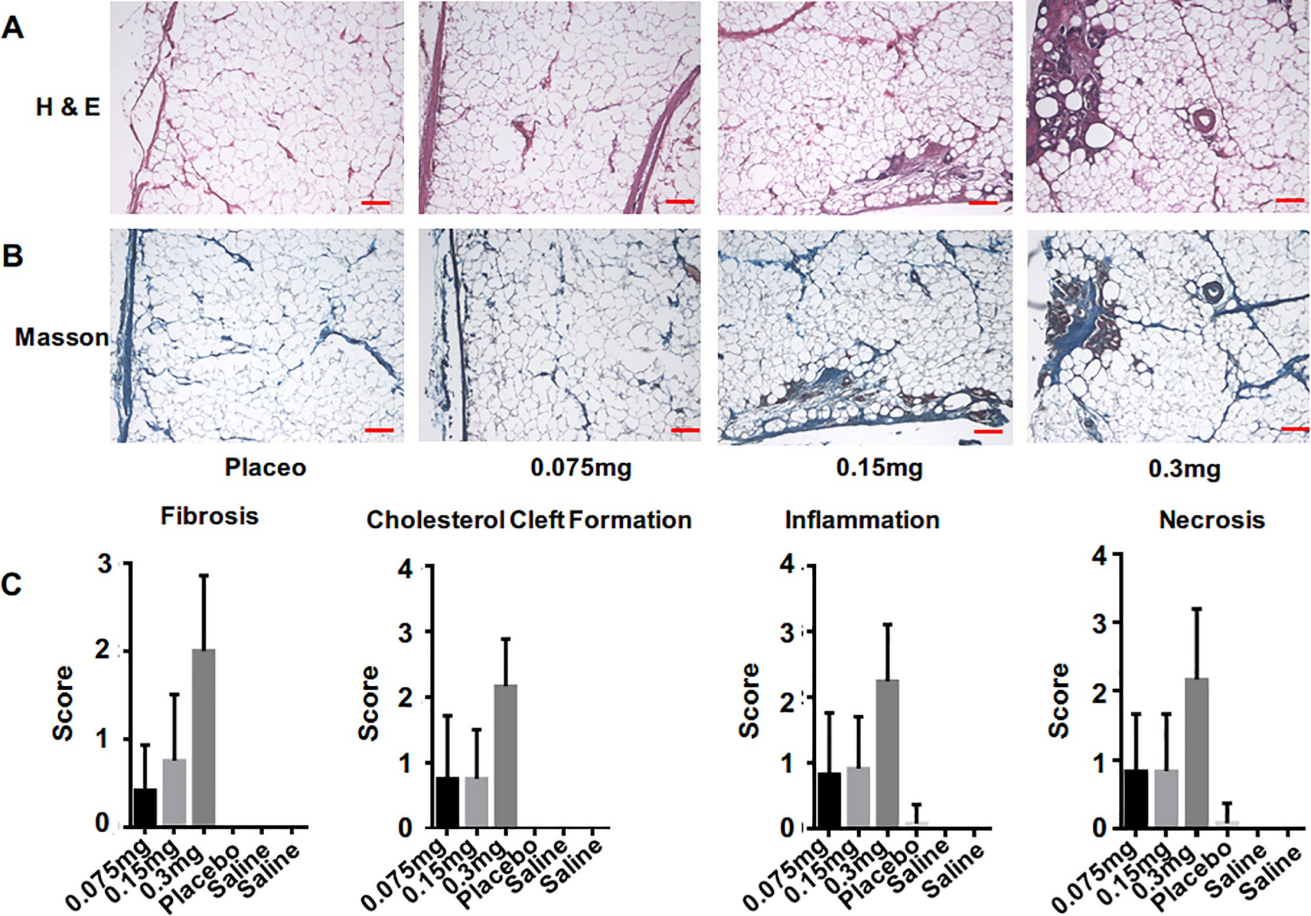
Histopathology evaluation results by H&E and masson staining. (A) H&E staining of adipose tissues from the placebo and 0.075/0.15/0.30 mg/injection areas. Scale bar = 0.5 mm. (B) Masson staining of adipose tissues from the placebo and 0.075/0.15/0.30 mg/injection areas. Scale bar = 0.5 mm. (C) The scores of histological parameters (fibrosis, cholesterol cleft, inflammation and necrosis). Data are presented as the mean ± SD, n = 12 (4 sections/area/minipig, 3 minipigs).

The adipose tissues obtained from the pharmacodynamic study were stained using H&E (Fig 5A) and Masson staining (Fig 5B), and the results showed marked destruction of adipose tissues and fibrosis.

The slides were evaluated by pathologists from PharmaLegacy Laboratories (Shanghai, China), who provided scores for fibrosis, cholesterol cleft, inflammation and necrosis (Fig 5C). While rColH(E451D) digests collagen, mainly collagen I and III [5], which are found in the adipocytes adhered to the ECM, the ruptured capillary vessels will induce hemorrhage, edema, adipocyte necrosis, inflammation and fibrosis. The scores showed a significant increase at the 0.3 mg dose, which suggests the safe range of rColH(E451D) administration by subcutaneous injection (S5 and S6 Tables).

## Discussion

The E451D mutation was established in wild-type ColH to decrease enzyme activity to better control side effects, and the purity was significantly increased to meet the requirements of medical application through a set of purification steps after fermentation in engineered *E*. *coli*. Importantly, the stability of rColH(FM) & rColH(WT) as determined by SDS-PAGE and enzyme activity analysis remained consistent after storage at 4°C or RT for 8 weeks, which suggests that the E451D mutation of the catalytic domain for ColH does not affect the stability.

Afterwards, an *E*. *coli* fermentation system and a purification method consisting of five chromatography steps were established to improve the purity of the mutant collagenase protein. SDS-PAGE, SEC, CE-SDS and Q-TOF-MS were used to prove the high purity of the protein (rColH(E451D)).

Moreover, the kinetic parameters of four kinds of enzymes (mutant or wild-type, with or without His tag) were analyzed. Compared with rColH(WT), rColH(FM) had similar *K*_*m*_ but decreased specific activity and *k*_*ca*t._ The lower the *K*_*m*_ value is, the higher the affinity to the substrate; the lower the *k*_*ca*t_ value is, the lower the catalytic efficiency. The above result indicates that the affinity of ColH(FM) to the substrate was not impaired, but the cleavage of collagen became much slower and milder, which means fewer side effects such as hemorrhage after the administration of mutant ColH. The results of rColH(E451D) and USP ColH were similar to those of rColH(FM) and rColH(WT); the *K*_*m*_ value of rColH(E451D) was significantly lower than that of USP ColH, which suggests that rColH(E451D) can bind substrates more easily than USP ColH.

To date, few studies have focused on the medical application of collagenase in adipolysis. Therefore, we used ColH(E451D) to cleave the collagen of ECM in adipose tissues and to remove local adipose by inducing adipocyte apoptosis. Before the pharmacodynamics study, we first investigated the enzyme kinetics of rColH(E451D) in *ob/ob* mice through injection into their inguinal adipose tissues. The results suggested that rColH(E451D) was inactivated *in vivo* within 24 h (S1 Fig). It is important that mutant ColH (rColH(E451D)) can be rapidly inactivated in blood through inhibition by serum proteins [25] because this makes subcutaneous injection safe and causes little systemic toxicity. Afterwards, the adipolysis effect of rColH(FM) and rColH(E451D) was proven *in vitro* and *in vivo*. Both results indicated that the adipolysis effect was dose-dependent. All doses of rColH(E451D) (0.075–0.3 mg) were effective for adipolysis *in vivo*, which is consistent with the mechanism of action (MOA) of collagenase. After rColH(E451D) treatment, adipose necrosis, inflammation, cholesterol cleft and fibrosis were observed in adipose tissues. Four weeks of recovery did not seem to be long enough, and the recovery period will be prolonged in the following study. Adipose necrosis and cholesterol cleft formation are regarded as evidence of adipocyte apoptosis. In contrast to the other three effects, fibrosis is a normal physiological response to tissue repair. Fibroblasts in adipose tissues will accumulate and cause collagen to reconstruct ECM [26]. Therefore, the fibrosis of adipose tissues is thought to be a local skin reaction instead of an adverse effect in this case.

## Conclusion

The E451D mutation does not influence the stability of the enzyme. Fermentation of *E*. *coli* BL21(DE3) and further purification help rColH(E451D) meet purity requirements for medical applications after five chromatography purification steps. The kinetic study of four kinds of enzymes (mutant or wild-type, with or without His tag) suggests that compared with USP ColH, rColH(E451D) has increased affinity to collagen but digests substrates more slowly, which will produce milder enzymolysis and induce fewer or slighter side effects *in vivo*. The efficacy study of rColH(E451D) on the back adipose tissues of minipigs showed that it exerts dose-dependent effect and that the side effects at specific doses are controllable and recoverable. In summary, our study provides proof-of-principle evidence that rColH(E451D) can be developed into an alternative treatment for local adipose removal in adults. Further investigations including clinical safety and efficacy studies will be conducted in the future.

## Supporting information

S1 Fig Enzyme activity kinetics curve of rColH(E451D) *in vivo* (*ob/ob* Mice). (DOCX)

S2 Fig Body weights of the minipigs used in the pharmacodynamics study. (DOCX)

S1 File Kinetic assay for rColH(WT). (PDF)

S2 File Kinetic assay for rColH(FM). (PDF)

S3 File Kinetic assay for USP ColH. (PDF)

S4 File Kinetic assay for rColH(E451D). (PDF)

S1 Table Specific activity of ColH. (DOCX)

S2 Table Kinetic parameters of wild-type and mutant ColH. (DOCX)

S3 Table Ratio of kinetic parameters. (DOCX)

S4 Table Mean relative thickness of each area in the pharmacodynamics study as determined by ultrasonography. (DOCX)

S5 Table Histopathological scoring results of each area in the pharmacodynamics study. (DOCX)

S6 Table Detailed histopathology scoring results of each area for each animal in pharmacodynamics study. (DOCX)
