# Supplementary figures and images for: Safe and effective subcutaneous adipolysis in minipigs by a collagenase derivative
Source: PLoS One. 2019 Dec 31;14(12):e0227202. doi: 10.1371/journal.pone.0227202 (PMC6938318; doi:10.1371/journal.pone.0227202)

S1 Fig. Enzyme activity kinetics curve of rColH(E451D) *in vivo* (*ob/ob* mice)


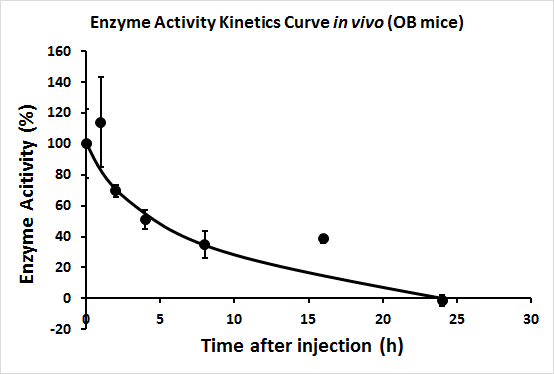

Supplement: S1 Fig — (DOCX) [file pone.0227202.s001.docx]
