# Supplementary material for: Safe and effective subcutaneous adipolysis in minipigs by a collagenase derivative
Source: PLoS One. 2019 Dec 31;14(12):e0227202. doi: 10.1371/journal.pone.0227202 (PMC6938318; doi:10.1371/journal.pone.0227202)
Supplement: S2 Fig — (DOCX) [file pone.0227202.s002.docx]

S2 Fig. Body weights of the minipigs used in pharmacodynamics study
