# Supplementary material for: Safe and effective subcutaneous adipolysis in minipigs by a collagenase derivative
Source: PLoS One. 2019 Dec 31;14(12):e0227202. doi: 10.1371/journal.pone.0227202 (PMC6938318; doi:10.1371/journal.pone.0227202)
Supplement: S2 File — (PDF) [file pone.0227202.s004.pdf]

STRENDA DB

Experimental data fact sheet

This document provides all functional enzyme data that were obtained under the given experimental conditions, entered into STRENDA DB and assigned to the unambiguous SRN shown in the first line. This document can be submitted together with the corresponding manuscript to a journal at one's own option.

Experiment Title	Kinetic assay for rColH(FM)
Strenda ID	SOAV35
DOI	10.22011/strenda_db.SOAV35

Manuscript Title	Safe and Effective Subcutaneous Adipolysis in Minipigs by a Collagenase Derivative
Authors	Chen FD, Du G, Shih MS, Yuan HJ, Bao P, Shi S, Cang Y, Zhang Z.

Methodology	An in vitro enzyme activity assay is conducted according to USP <89.2> Collagenase II, using a synthetic peptide (4-Phenylazobenzyloxycarbonyl(PZ)-Pro-Leu-Gly-Pro-D-Arg, trifluoroacetate salt, Sigma) as substrate. The test articles are diluted to 0.2-0.3 mg/mL by 0.1 M Tris, PH7.1. One Unit will release the equivalent of 1 µmol of PZ-Pro-Leu from PZ-Pro-Leu-Gly-Pro-D-Arg per minute under the conditions of the assay. For enzyme kinetics studies, the assay is carried out with various concentrations of the substrate (0.1, 0.15, 0.2, 0.25, 0.33, 0.5mg/mL). Km and Vmax values were obtained by Linear regression of Microsoft Excel (Lineweaver-Burk plot analysis of 1/V versus 1/[S]). kcat = Vmax / [E], E=millimolar concentration of enzyme in the reaction system. Values are means ± standard deviations of triplicate measurements using Graphpad 6.0. Protein concentrations are determined by the method of ultraviolet spectrophotometry. All the assays are tested in triplicate.

Protein

Protein Description

Is the protein data registered in UniProtKB?	no
UniProtKB AC	N.A.
Protein Name	Recombinant Mutant Collagenase H with 6x His Tag (rColH(FM))
Sequence	MVQNESKRYTVSYLKTLNYYDLVDLLVKTEIENLPDLFQYSSDAKEFYGNKTRMSFIMD<br>EIGRRAPQYTEIDHKGIPTLVEVVRAGFYLGFHNKELNEINKRSFKERVIPSILAIQKN<br>PNFKLGTEVQDKIVSATGLLAGNETAPPEVVNNFTPILQDCIKNIDRYALDDLKSKALF<br>NVLAAPTYDITEYLRATKEKPENTPWYGKIDGFINELKKLALYGKINDNNSWIIDNGIY<br>HIAPLGKLHSNNKIGIETLTEVMKVYPYLSMQHLQSADQIKRHYDSKDAEGNKIPLDKF<br>KKEGKEKYCPKTYTFDDGKVIIKAGARVEEEKVKRLYWASKEVNSQFFRVYGIDKPLEE<br>GNPDDILTMVIYNSPEEYKLNSVLYGYDTNNGGMYIEPEGTFFTYEREAQESTYTLEEL<br>FRHEYTHYLQGRYAVPGQWGRTKLYDNDRLTWYEEGGADLFAGSTRTSGILPRKSIVSN<br>IHNTTRNNRYKLSDTVHSKYGASFEFYNYACMFMDYMYNKDMGILNKLNDLAKNNDVDG<br>YDNYIRDLSSNYALNDKYQDHMQERIDNYENLTVPFVADDYLVRHAYKNPNEIYSEISE<br>VAKLKDAKSEVKKSQYFSTFTLRGSYTGGASKGKLEDQKAMNKFIDDSLKKLDTYSWSG<br>YKTLTAYFTNYKVDSSNRVTYDVVFHGYLPNEGDSKNSLPYGKINGTYKGTEKEKIKFS<br>SEGSFDPDGKIVSYEWDFGDGNKSNEENPEHSYDKVGTYTVKLKVTDDKGESSVSTTTA<br>EIKDLSENKLPVIYMHVPKSGALNQKVVFYGKGTYDPDGSIAGYQWDFGDGSDFSSEQN<br>PSHVYTKKGEYTVTLRVMDSSGQMSEKTMKIKITDPVYPIGTEKEPNNSKETASGPIVP<br>GIPVSGTIENTSDQDYFYFDVITPGEVKIDINKLGYGGATWVVYDENNNAVSYATDDGQ<br>NLSGKFKADKPGRYYIHLYMFNGSYMPYRINIEGSVGRLEHHHHHH

Protein Sequence Modifications

Does the protein contain any sequence modification(s) in comparision to that of the UniProtKB entry?	no

Posttranslational Modifications (PTM)

Was the protein chemically modified after its translation?	no

Protein Source

Was the protein expressed from a plasmid?	yes

Comments	expressed in E. coli BL21 (DE3) using pET-30a(+)
Expression system	Escherichia coli
Organism as in UniProtKB	Clostridium histolyticum

Protein Reaction

Comment on Protein Reaction	The enzyme activity assay is conducted according to USP <89.2> Collagenase II, using a synthetic peptide (4-Phenylazobenzyloxycarbonyl(PZ)-Pro-Leu-Gly-Pro-D-Arg, trifluoroacetate salt, Sigma) as substrate.

Datasets

Dataset name : kinetic mechanism of rColH(FM)

Assay Conditions

Small Compounds

Role	CID	Name	Value
Substrate		4-Phenylazobenzyloxycarbonyl(PZ)-Pro-Leu-Gly-Pro-D-Arg, trifluoroacetate salt	0.103 - .515 mM
Buffer	6503	Trometamol	0.1 M

Protein Concentration

Concentration of the asssayed protein	152.857 nM
How was the protein concentratrion measured	extinction coefficient (A280 1%/cm) for collagenase, 1.4

Physical Properties

pH	7.1
Temperature	25.0 °C

Results

Kinetic parameters

Role	Name	Value	
Substrate	4-Phenylazobenzyloxycarbonyl(PZ)-Pro-Leu-Gly-Pro-D-Arg, trifluoroacetate salt	K_m	0.77 (+/-) 0.09 mM
		k_{cat}	3.57 (+/-) 0.33 s^{-1}
		V	0.033 (+/-) 0.003 mM min^{-1}
		k_{cat}/K_m	4.64 (+/-) 0.12 $mM^{-1}s^{-1}$
