# Supplementary material for: Safe and effective subcutaneous adipolysis in minipigs by a collagenase derivative
Source: PLoS One. 2019 Dec 31;14(12):e0227202. doi: 10.1371/journal.pone.0227202 (PMC6938318; doi:10.1371/journal.pone.0227202)
Supplement: S3 File — (PDF) [file pone.0227202.s005.pdf]

# STRENDA DB

## Experimental data fact sheet

This document provides all functional enzyme data that were obtained under the given experimental conditions, entered into STRENDA DB and assigned to the unambiguous SRN shown in the first line. This document can be submitted together with the corresponding manuscript to a journal at one's own option.

| | |
|---|---|
| Experiment Title | Kinetic assay for USP ColH |
| Strenda ID | HZTRM7 |
| DOI | 10.22011/strenda_db.HZTRM7 |

| | |
|---|---|
| Manuscript Title | Safe and Effective Subcutaneous Adipolysis in Minipigs by a Collagenase Derivative |
| Authors | Chen FD, Du G, Shih MS, Yuan HJ, Bao P, Shi S, Cang Y, Zhang Z. |

| | |
|---|---|
| Methodology | An in vitro enzyme activity assay is conducted according to USP <89.2> Collagenase II, using a synthetic peptide (4-Phenylazobenzyloxycarbonyl(PZ)-Pro-Leu-Gly-Pro-D-Arg, trifluoroacetate salt, Sigma) as substrate. The test articles are diluted to 0.2-0.3 mg/mL by 0.1 M Tris, PH7.1. One Unit will release the equivalent of 1 µmol of PZ-Pro-Leu from PZ-Pro-Leu-Gly-Pro-D-Arg per minute under the conditions of the assay. For enzyme kinetics studies, the assay is carried out with various concentrations of the substrate (0.1, 0.15, 0.2, 0.25, 0.33, 0.5mg/mL). Km and Vmax values were obtained by Linear regression of Microsoft Excel (Lineweaver-Burk plot analysis of 1/V versus 1/[S]). kcat = Vmax / [E], E=millimolar concentration of enzyme in the reaction system. Values are means ± standard deviations of triplicate measurements using Graphpad 6.0. Protein concentrations are determined by the method of ultraviolet spectrophotometry. All the assays are tested in triplicate. |

# Protein

### Protein Description

| | |
|---|---|
| Is the protein data registered in UniProtKB? | no |
| UniProtKB AC | N.A. |
| Protein Name | USP ColH |
| Sequence | VQNESKRYTVSYLKTLNYYDLVDLLVKTEIENLPDLFQYSSDAKEFYGNKTRMSFIMDE<br>IGRRAPQYTEIDHKGIPTLVEVVRAGFYLGFHNKELNEINKRSFKERVIPSILAIQKNP<br>NFKLGTEVQDKIVSATGLLAGNETAPPEVVNNFTPILQDCIKNIDRYALDDLKSKALFN<br>VLAAPTYDITEYLRATKEKPENTPWYGKIDGFINELKKLALYGKINDNNSWIIDNGIYH<br>IAPLGKLHSNNKIGIETLTEVMKVYPYLSMQHLQSADQIKRHYDSKDAEGNKIPLDKFK<br>KEGKEKYCPKTYTFDDGKVIIKAGARVEEEKVKRLYWASKEVNSQFFRVYGIDKPLEEG<br>NPDDILTMVIYNSPEEYKLNSVLYGYDTNNGGMYIEPEGTFFTYEREAQESTYTLEELF<br>RHEYTHYLQGRYAVPGQWGRTKLYDNDRLTWYEEGGAELFAGSTRTSGILPRKSIVSNI<br>HNTTRNNRYKLSDTVHSKYGASFEFYNYACMFMDYMYNKDMGILNKLNDLAKNNDVDGY<br>DNYIRDLSSNYALNDKYQDHMQERIDNYENLTVPFVADDYLVRHAYKNPNEIYSEISEV<br>AKLKDAKSEVKKSQYFSTFTLRGSYTGGASKGKLEDQKAMNKFIDDSLKKLDTYSWSGY<br>KTLTAYFTNYKVDSSNRVTYDVVFHGYLPNEGDSKNSLPYGKINGTYKGTEKEKIKFSS<br>EGSFDPDGKIVSYEWDFGDGNKSNEENPEHSYDKVGTYTVKLKVTDDKGESSVSTTTAE<br>IKDLSENKLPVIYMHVPKSGALNQKVVFYGKGTYDPDGSIAGYQWDFGDGSDFSSEQNP<br>SHVYTKKGEYTVTLRVMDSSGQMSEKTMKIKITDPVYPIGTEKEPNNSKETASGPIVPG<br>IPVSGTIENTSDQDYFYFDVITPGEVKIDINKLGYGGATWVVYDENNNAVSYATDDGQN<br>LSGKFKADKPGRYYIHLYMFNGSYMPYRINIEGSVGR |

### Protein Sequence Modifications

| | |
|---|---|
| Does the protein contain any sequence modification(s) in comparision to that of the UniProtKB entry? | no |

### Posttranslational Modifications (PTM)

| | |
|---|---|
| Was the protein chemically modified after its translation? | no |

### Protein Source

| | |
|---|---|
| Was the protein expressed from a plasmid? | no |

| | |
|---|---|
| Organism as in UniProtKB | Clostridium histolyticum |

**Protein Reaction**

| | |
|---|---|
| Comment on Protein Reaction | The enzyme activity assay is conducted according to USP <89.2> Collagenase II, using a synthetic peptide (4-Phenylazobenzyloxycarbonyl(PZ)-Pro-Leu-Gly-Pro-D-Arg, trifluoroacetate salt, Sigma) as substrate. |

# Datasets

## *Dataset name : kinetic mechanism of USP ColH*

### Assay Conditions

***Small Compounds***

| Role | CID | Name | Value |
|---|---|---|---|
| Buffer | 6503 | Trometamol | 0.1 M |
| Substrate | | 4-Phenylazobenzyloxycarbonyl(PZ)-Pro-Leu-Gly-Pro-D-Arg, trifluoroacetate salt | 0.103 - .515 mM |

***Protein Concentration***

| | |
|---|---|
| Concentration of the asssayed protein | 7.429 nM |
| How was the protein concentratrion measured | extinction coefficient ( A280 1%/cm) for collagenase, 1.4 |

***Physical Properties***

| | |
|---|---|
| pH | 7.1 |
| Temperature | 25.0 °C |

### Results

***Kinetic parameters***

| Role | Name | Value | |
|---|---|---|---|
| Substrate | 4-Phenylazobenzyloxycarbonyl(PZ)-Pro-Leu-Gly-Pro-D-Arg, trifluoroacetate salt | $K_m$ | 0.7 (+/-) 0.08 mM |
| | | $k_{cat}$ | 62.12 (+/-) 4.8 $s^{-1}$ |
| | | $V$ | 0.028 (+/-) 0.002 mM $min^{-1}$ |
| | | $k_{cat}/K_m$ | 88.85 (+/-) 3.36 $mM^{-1}s^{-1}$ |
