# Supplementary material for: Safe and effective subcutaneous adipolysis in minipigs by a collagenase derivative
Source: PLoS One. 2019 Dec 31;14(12):e0227202. doi: 10.1371/journal.pone.0227202 (PMC6938318; doi:10.1371/journal.pone.0227202)
Supplement: S1 Table — (DOCX) [file pone.0227202.s007.docx]

S1 Table. Specific activity of ColH

| Enzyme | U/mg | % (±SD %) of wild-type ColH activity |
| --- | --- | --- |
| USP ColH | 12 ± 1.2 | 9.13 ± 0.7 |
| rColH(E451D) | 1.1 ± 0.19 |  |
| rColH(WT) | 5.56 ± 0.22 | 13.3 ± 0.2 |
| rColH(FM) | 0.74 ± 0.02 |  |
