# Supplementary material for: Safe and effective subcutaneous adipolysis in minipigs by a collagenase derivative
Source: PLoS One. 2019 Dec 31;14(12):e0227202. doi: 10.1371/journal.pone.0227202 (PMC6938318; doi:10.1371/journal.pone.0227202)
Supplement: S2 Table — (DOCX) [file pone.0227202.s008.docx]

S2 Table. Kinetic parameters of the wild-type and mutant ColH

| **Enzyme** | ***k_cat_*(s^-1^)** | ***K_m_*(mM)** | ***k_cat_ / K_m_* (mM^-1^s^-1^)** |
| --- | --- | --- | --- |
| **USP ColH** | 62.12 ± 4.8 | 0.7 ± 0.08 | 88.85 ± 3.36 |
| **rColH(E451D)** | 3.33 ± 0.31 | 0.48 ± 0.07 | 6.95 ± 0.38 |
| **rColH(WT)** | 28.69 ± 0.81 | 0.85 ± 0.03 | 33.78 ± 0.24 |
| **rColH(FM)** | 3.57 ± 0.33 | 0.77 ± 0.09 | 4.64 ± 0.12 |
