# Supplementary material for: Safe and effective subcutaneous adipolysis in minipigs by a collagenase derivative
Source: PLoS One. 2019 Dec 31;14(12):e0227202. doi: 10.1371/journal.pone.0227202 (PMC6938318; doi:10.1371/journal.pone.0227202)
Supplement: S3 Table — (DOCX) [file pone.0227202.s009.docx]

S3 Table. Ratio of kinetic parameters

| **Enzyme** | **Ratio of *K_m_*** | **Ratio of *k_cat_*** |
| --- | --- | --- |
| **USP ColH / rColH(E451D)** | 1.45 | 18.65 |
| **rColH(WT) / rColH(FM)** | 1.1 | 8.03 |
