# Supplementary material for: Safe and effective subcutaneous adipolysis in minipigs by a collagenase derivative
Source: PLoS One. 2019 Dec 31;14(12):e0227202. doi: 10.1371/journal.pone.0227202 (PMC6938318; doi:10.1371/journal.pone.0227202)
Supplement: S4 Table — (DOCX) [file pone.0227202.s010.docx]

S4 Table. Mean relative thickness of each area in pharmacodynamics study by ultrasonography

| **Group** | | **Week 0**  **(%)** | **Week 1**  **(%)** | **Week 2**  **(%)** | **Week 3**  **(%)** | **Week 4**  **(%)** |
| --- | --- | --- | --- | --- | --- | --- |
| **Area A: 0.075 mg/point** | **Mean** | 100.00 | 100.13 | 96.63 | 91.03 | 90.18 |
|  | **SD** | 0.00 | 6.50 | 6.46 | 2.71 | 6.86 |
| **Area B: 0.15 mg/point** | **Mean** | 100.00 | 102.11 | 93.82 | 90.40 | 88.39 |
|  | **SD** | 0.00 | 10.77 | 2.81 | 2.90 | 2.97 |
| **Area C: 0.3 mg/point** | **Mean** | 100.00 | 107.32 | 90.74 | 83.53 | 85.32 |
|  | **SD** | 0.00 | 7.79 | 4.65 | 4.74 | 4.54 |
| **Area D: Placebo** | **Mean** | 100.00 | 103.91 | 107.11 | 106.17 | 110.33 |
|  | **SD** | 0.00 | 1.18 | 0.80 | 1.28 | 6.67 |
| **Area E: Saline** | **Mean** | 100.00 | 105.65 | 107.13 | 105.06 | 111.55 |
|  | **SD** | 0.00 | 3.47 | 5.96 | 5.70 | 7.93 |
| **Area F: Saline** | **Mean** | 100.00 | 99.83 | 103.95 | 103.65 | 113.39 |
|  | **SD** | 0.00 | 2.28 | 2.88 | 1.03 | 3.70 |
