# Supplementary material for: Safe and effective subcutaneous adipolysis in minipigs by a collagenase derivative
Source: PLoS One. 2019 Dec 31;14(12):e0227202. doi: 10.1371/journal.pone.0227202 (PMC6938318; doi:10.1371/journal.pone.0227202)
Supplement: S5 Table — (DOCX) [file pone.0227202.s011.docx]

S5 Table. Mean histopathology scoring results of each area in pharmacodynamics study

| **Group** | | **Adipose**  **Necrosis**  **(0-4)** | **Inflammation**  **(0-4)** | **Cholesterol Cleft Formation**  **(0-4)** | **Fibrosis**  **(0-4)** | **Hemorrhage (0-4)** |
| --- | --- | --- | --- | --- | --- | --- |
| **Area A: 0.075 mg/point** | **Mean** | 0.83 | 0.83 | 0.75 | 0.42 | 0.00 |
|  | **SD** | 0.83 | 0.94 | 0.97 | 0.51 | 0.00 |
| **Area B: 0.15 mg/point** | **Mean** | 0.83 | 0.92 | 0.75 | 0.75 | 0.00 |
|  | **SD** | 0.83 | 0.79 | 0.75 | 0.75 | 0.00 |
| **Area C: 0.3 mg/point** | **Mean** | 2.17 | 2.25 | 2.17 | 2.00 | 0.00 |
|  | **SD** | 1.03 | 0.87 | 0.72 | 0.85 | 0.00 |
| **Area D: Placebo** | **Mean** | 0.08 | 0.08 | 0.00 | 0.00 | 0.00 |
|  | **SD** | 0.29 | 0.29 | 0.00 | 0.00 | 0.00 |
| **Area E: Saline** | **Mean** | 0.00 | 0.00 | 0.00 | 0.00 | 0.00 |
|  | **SD** | 0.00 | 0.00 | 0.00 | 0.00 | 0.00 |
| **Area F: Saline** | **Mean** | 0.00 | 0.00 | 0.00 | 0.00 | 0.00 |
|  | **SD** | 0.00 | 0.00 | 0.00 | 0.00 | 0.00 |
