# Supplementary material for: Safe and effective subcutaneous adipolysis in minipigs by a collagenase derivative
Source: PLoS One. 2019 Dec 31;14(12):e0227202. doi: 10.1371/journal.pone.0227202 (PMC6938318; doi:10.1371/journal.pone.0227202)
Supplement: S6 Table — (DOCX) [file pone.0227202.s012.docx]

S6 Table. Detailed histopathology scoring results of each area for each animal in pharmacodynamics study

| **An ID** | **Site ID** | **Adipose Necrosis (0-4)** | **Inflammation**  **(0-4)** | **Cholesterol**  **Cleft Formation (0-4)** | **Fibrosis**  **(0-4)** | **Hemorrhage**  **(0-4)** |
| --- | --- | --- | --- | --- | --- | --- |
| 0891 | A-1 | 2 | 1 | 0 | 0 | 0 |
| 0891 | A-2 | 1 | 1 | 1 | 1 | 0 |
| 0891 | A-3 | 0 | 0 | 0 | 0 | 0 |
| 0891 | A-4 | 0 | 0 | 0 | 0 | 0 |
| 0891 | B-1 | 3 | 2 | 1 | 0 | 0 |
| 0891 | B-2 | 1 | 1 | 1 | 1 | 0 |
| 0891 | B-3 | 0 | 0 | 0 | 0 | 0 |
| 0891 | B-4 | 0 | 0 | 0 | 0 | 0 |
| 0891 | C-1 | 3 | 2 | 1 | 1 | 0 |
| 0891 | C-2 | 1 | 2 | 2 | 1 | 0 |
| 0891 | C-3 | 2 | 2 | 2 | 2 | 0 |
| 0891 | C-4 | 3 | 3 | 3 | 3 | 0 |
| 0891 | D-1 | 0 | 0 | 0 | 0 | 0 |
| 0891 | D-2 | 0 | 0 | 0 | 0 | 0 |
| 0891 | D-3 | 0 | 0 | 0 | 0 | 0 |
| 0891 | D-4 | 0 | 0 | 0 | 0 | 0 |
| 0891 | E-1 | 0 | 0 | 0 | 0 | 0 |
| 0891 | E-2 | 0 | 0 | 0 | 0 | 0 |
| 0891 | E-3 | 0 | 0 | 0 | 0 | 0 |
| 0891 | E-4 | 0 | 0 | 0 | 0 | 0 |
| 0891 | F-1 | 0 | 0 | 0 | 0 | 0 |
| 0891 | F-2 | 0 | 0 | 0 | 0 | 0 |
| 0891 | F-3 | 0 | 0 | 0 | 0 | 0 |
| 0891 | F-4 | 0 | 0 | 0 | 0 | 0 |
| 1202 | A-1 | 2 | 2 | 2 | 0 | 0 |
| 1202 | A-2 | 0 | 0 | 0 | 0 | 0 |
| 1202 | A-3 | 0 | 0 | 0 | 0 | 0 |
| 1202 | A-4 | 0 | 0 | 0 | 0 | 0 |
| 1202 | B-1 | 0 | 0 | 0 | 0 | 0 |
| 1202 | B-2 | 1 | 1 | 1 | 1 | 0 |
| 1202 | B-3 | 0 | 0 | 0 | 0 | 0 |
| 1202 | B-4 | 1 | 1 | 1 | 1 | 0 |
| 1202 | C-1 | 2 | 2 | 3 | 1 | 0 |
| 1202 | C-2 | 1 | 2 | 2 | 2 | 0 |
| 1202 | C-3 | 1 | 1 | 1 | 1 | 0 |
| 1202 | C-4 | 1 | 1 | 2 | 2 | 0 |
| 1202 | D-1 | 0 | 0 | 0 | 0 | 0 |
| 1202 | D-2 | 0 | 0 | 0 | 0 | 0 |
| 1202 | D-3 | 0 | 0 | 0 | 0 | 0 |
| 1202 | D-4 | 0 | 0 | 0 | 0 | 0 |
| 1202 | E-1 | 0 | 0 | 0 | 0 | 0 |
| 1202 | E-2 | 0 | 0 | 0 | 0 | 0 |
| 1202 | E-3 | 0 | 0 | 0 | 0 | 0 |
| 1202 | E-4 | 0 | 0 | 0 | 0 | 0 |
| 1202 | F-1 | 0 | 0 | 0 | 0 | 0 |
| 1202 | F-2 | 0 | 0 | 0 | 0 | 0 |
| 1202 | F-3 | 0 | 0 | 0 | 0 | 0 |
| 1202 | F-4 | 0 | 0 | 0 | 0 | 0 |
| 1203 | A-1 | 2 | 3 | 3 | 1 | 0 |
| 1203 | A-2 | 1 | 1 | 1 | 1 | 0 |
| 1203 | A-3 | 1 | 1 | 1 | 1 | 0 |
| 1203 | A-4 | 1 | 1 | 1 | 1 | 0 |
| 1203 | B-1 | 1 | 1 | 0 | 1 | 0 |
| 1203 | B-2 | 1 | 1 | 1 | 1 | 0 |
| 1203 | B-3 | 1 | 2 | 2 | 2 | 0 |
| 1203 | B-4 | 1 | 2 | 2 | 2 | 0 |
| 1203 | C-1 | 4 | 4 | 3 | 3 | 0 |
| 1203 | C-2 | 3 | 3 | 2 | 2 | 0 |
| 1203 | C-3 | 3 | 3 | 3 | 3 | 0 |
| 1203 | C-4 | 2 | 2 | 2 | 3 | 0 |
| 1203 | D-1 | 1 | 1 | 0 | 0 | 0 |
| 1203 | D-2 | 0 | 0 | 0 | 0 | 0 |
| 1203 | D-3 | 0 | 0 | 0 | 0 | 0 |
| 1203 | D-4 | 0 | 0 | 0 | 0 | 0 |
| 1203 | E-1 | 0 | 0 | 0 | 0 | 0 |
| 1203 | E-2 | 0 | 0 | 0 | 0 | 0 |
| 1203 | E-3 | 0 | 0 | 0 | 0 | 0 |
| 1203 | E-4 | 0 | 0 | 0 | 0 | 0 |
| 1203 | F-1 | 0 | 0 | 0 | 0 | 0 |
| 1203 | F-2 | 0 | 0 | 0 | 0 | 0 |
| 1203 | F-3 | 0 | 0 | 0 | 0 | 0 |
| 1203 | F-4 | 0 | 0 | 0 | 0 | 0 |

Histopathology grading:

0 = normal, no obvious cell or tissue reaction/damage

1 = slight or mild changes associated with cell or tissue reaction/damage

2 = moderate changes associated with cell or tissue reaction/damage

3 = moderate to severe or marked changes associated with cell or tissue reaction/damage

4 = marked changes associated with cell or tissue reaction/damage
